# A PP2A-mtATR-tBid axis links DNA damage-induced CIP2A degradation to apoptotic dormancy and therapeutic resistance in PDAC

**DOI:** 10.1016/j.canlet.2025.217790

**Published:** 2025-05-10

**Authors:** Yibo Luo, Himadri Biswas, Yetunde Makinwa, Shi-He Liu, Zizheng Dong, Jing-Yuan Liu, Jian-Ting Zhang, Yue Zou

**Affiliations:** Department of Cell and Cancer Biology, University of Toledo College of Medicine and Life Sciences, Toledo, OH, 43614, USA

**Keywords:** PDAC drug resistance, DNA damage, Apoptotic dormancy, Mitochondrial ATR-tBid complex, CIP2A, And LB-100

## Abstract

DNA damage-based drugs are widely used in cancer therapy, yet resistance remains a major challenge. In this study, we uncovered a non-DNA repair mechanism contributing to resistance in pancreatic ductal adenocarcinoma (PDAC). We show that in gemcitabine-resistant PDAC cells, CIP2A undergoes ubiquitin-mediated degradation, resulting in enhanced PP2A phosphatase activity. This leads to the dephosphorylation of ATR at Ser428 in the cytoplasm, promoting the formation of the prolyl *cis*-isomeric form of ATR at its Ser428-Pro429 motif. The resulting *cis*-ATR functions as a mitochondria-targeted antiapoptotic protein (mtATR). Surprisingly, resistant PDAC cells paradoxically accumulated both mtATR and proapoptotic tBid at the mitochondria, forming a stable mtATR-tBid complex that induces a state of apoptotic dormancy. Disrupting this complex, either with the PP2A inhibitor LB-100 or a cytoplasmic ATR-specific antibody, reactivates the pre-accumulated tBid and restores apoptosis in resistant PDAC cells. In an orthotopic PDAC mouse model, LB-100 alone significantly inhibit gemcitabine-resistant tumor growth by disrupting the mtATR-tBid complex. These findings reveal a previously unrecognized mechanism of resistance to DNA damage-based therapies and identify a novel action mechanism of LB-100, characterized by the CIP2A degradation-mediated PP2A-mtATR-tBid axis. By targeting mtATR-tBid-mediated apoptotic dormancy, this strategy offers a promising approach to restore apoptotic sensitivity in drug-resistant cancers.

## 1. Introduction

Anticancer drugs that induce DNA damage or impair DNA repair pathways have been widely used to treat malignancies, primarily by triggering apoptosis. However, acquired drug resistance often develops after prolonged chemotherapy treatment, as cancer cells adapt a more plastic condition to survive despite genome instability [1–5]. DNA repair pathways are well-established as key contributors to cancer drug resistance. In this study, however, we present a novel mechanism of DNA damage-induced cancer drug resistance, which extends beyond DNA repair and is characterized by apoptotic dormancy at the mitochondria.

Cancer cells can survive through various mechanisms, such as metabolic reprogramming, insufficient drug accumulation, immunosuppression, increased DNA repair, etc. [6–8]. Among these, disrupting the balance of apoptotic-related BCL-2 family proteins has emerged as a promising strategy for cancer treatment. In general, the activity of anti-apoptotic pathway and overexpression of Bcl-2 in response to apoptotic stimuli have garnered significant interest in cancer therapy [9–12]. In these cells, the anti-apoptotic pathway is typically inhibited or downregulated, while proapoptotic proteins are overexpressed. However, these treatments have shown limited success. A prominent example of resistance is senescence. Senescent cells exhibit strong resistance to apoptosis, despite the downregulation of Bcl-2, Bcl-XL and Mcl-1 [13,14]. Meanwhile proapoptotic factors are overexpressed, and DNA damage accumulates significantly [15–17]. The current study unveils a novel mechanism of apoptosis dormancy that contributes to DNA damage-based cancer drug resistance. Awakening this dormancy “to reactivate proapoptotic signaling may present a new strategic approach for overcoming cancer drug resistance.

Protein phosphatase 2A (PP2A) is a major serine/threonine phosphatase that plays a crucial role in regulating various cellular processes. It is a heterotrimeric complex of three subunits: the catalytic subunit C (PP2A-C), the structural subunit A (scaffold), and the regulatory subunit B, which has multiple isoforms. The B subunit determines PP2A’s substrate specificity, cellular localization, tissue specificity, and functions, depending on the specific isoform present within the heterotrimer [18–21]. In cancer cells, cancerous inhibitor of protein phosphatase 2A (CIP2A) inhibits PP2A activity by hijacking the B subunit of PP2A complex, forming a CIP2A-subunit B-PP2Ac trimer by expulsing subunit A to interrupt PP2A holoenzyme activity [22,23]. Preclinical studies have shown that reactivating PP2A can efficiently prevent tumor development and progression [24–27], and CIP2A’s inhibitory role in cancer progression by suppressing PP2A activity has also been highlighted [28,29]. However, the role of PP2A and CIP2A in cellular processes might not be simply pinned down to tumor suppression given their involvement in all aspects of cellular signaling pathways, particularly due to the diverse isoforms of the B regulatory subunits [28–34].

We previously demonstrated that upon DNA damage, a prolyl *cis*-isomeric form of cytoplasmic *ataxia telangiectasia* and Rad3 related (ATR), promoted by PP2A, translocated to the mitochondria, where it binds to and inhibits the proapoptotic tBid [35]. PP2A dephosphorylates ATR at Ser428, inhibiting the prolyl isomerase Pin1 and promoting the formation of the mitochondria-targeted ATR isoform (mtATR) [36,37]. Knocking down PP2A by siRNA restores phosphorylation of ATR at Ser428 and removes the mtATR from the mitochondria.

In this study, we present evidence for a novel mechanism of DNA damage-induced cancer drug resistance. This mechanism involves the accumulation of an antiapoptotic and proapoptotic mtATR-tBid complex, which mediates apoptotic dormancy at mitochondria, contributing to drug resistance. This resistance is directly regulated by CIP2A-modulated PP2A activity. Specifically, tBid is enriched at the mitochondria of gemcitabine-resistant pancreatic ductal adenocarcinoma (PDAC) cells, but the cells remain apoptosis-dormant due to the binding of mtATR to tBid. Inhibiting PP2A activity using using LB-100, a candidate drug currently in clinical trials LB-100 or a cytoplasmic ATR-specific antibody eliminates mitochondrial ATR and reactivates apoptosis from the dormant state, leading to cell death in PDAC cells. Further investigation of the mechanisms reveals that CIP2A undergoes substantial ubiquitin-mediated degradation in gemcitabine-resistant PDAC cells, whereas it remains enriched in parental PDAC cells. Our results highlight the crucial role of the CIP2A-PP2A-mitochondrial ATR-tBid axis in driving drug resistance in PDAC, likely extending to other cancers, through the induction of apoptotic dormancy. Targeting this pathway may offer a promising strategy to overcome resistance to DNA damage-based therapies.

## 2. Results

### 2.1. Establishment and characterization of gemcitabine-resistant pancreatic cancer cell lines

**Fig. 1.**
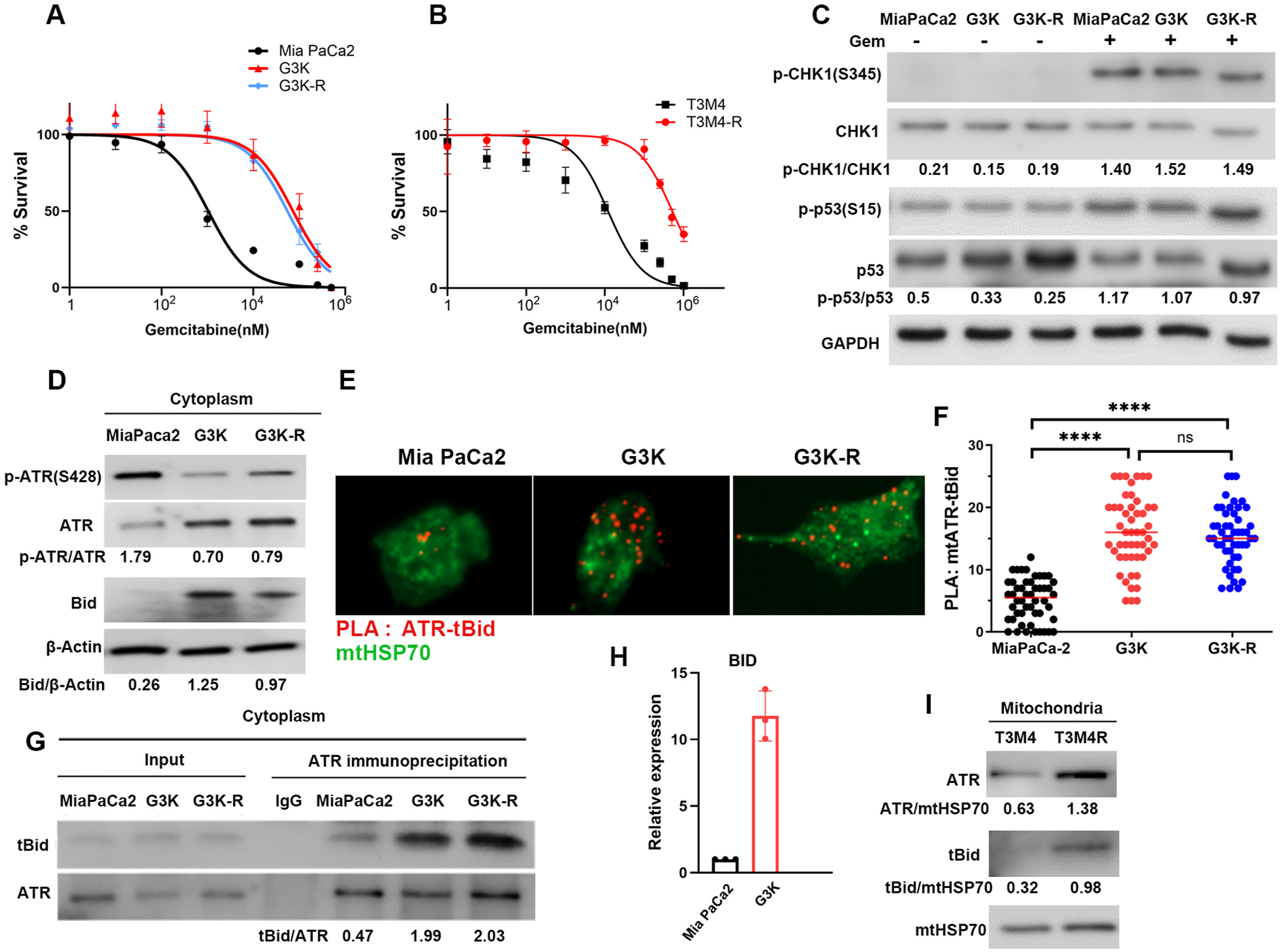
-accumulation of antiapoptotic mtATR and proapoptotic tBid, and their complex at mitochondria in gemcitabine-resistant PDAC cells. **Co** (A) Representative cell survival curve after gemcitabine treatment at various concentrations in Mia PaCa-2, G3K, and G3K-Rev(G3K-R) cells. The IC50 of Gemcitabine in the three cell lines were 1.04 ± 0.12 μM, 73.0 ± 9.28 μM and 55.4 ± 4.78 μM separately, generated from three independent experiments. (B) Representative cell survival curves after gemcitabine treatment at various concentrations in T3M4 and T3M4-R cells. IC50 of gemcitabine in the two cell lines were 12.16 ± 2.58 μM and 521.35 ± 34.13 μM, generated from three independent experiments. (C) DNA damage response pathway detection in Mia PaCa-2, G3K and G3K-R cells after gemcitabine treatment. 1 μM gemcitabine was applied to cells for 12 h. Whole cell lysates were subjected to Western blot. The mean values under each panel were from 3 independent repeats. (D) Cytoplasm fraction was isolated from Mia PaCa-2, G3K, and G3K-R cells and was subjected to Western blot. Total ATR, phosphor-ATR(S428) and Bid were detected. β-Actin was used as loading control. The mean values under each panel were from 3 independent repeats. (E) Proximity ligand assay (PLA) result showing the mtATR/tBid complex on mitochondria. The red foci indicated the mtATR-tBid complex. (F) Quantification of PLA foci at mitochondria in tested cell lines (n = 50). (G) Immunoprecipitation results showed the increased ATR/tBid binding in G3K and G3K-R cells but not in Mia PaCa-2 cells using cytoplasm fraction. The mean values under each panel were from 3 independent repeats. (H) qRT-PCR detecting the significant upregulation of Bid mRNA at the transcription level. (I) Mitochondria fraction was isolated from T3M4 and T3M4-R cells and was subjected to Western blot. ATR was significantly accumulated on mitochondria in T3M4-R cells. Three independent measurements were performed.

To gain insight into the mechanisms for the DNA damage-based cancer drug resistance, PDAC cell lines Mia PaCa-2 and T3M4 were cultured under gradually increased gemcitabine selection pressure for over 6 months to generate drug resistant-cell clones [38]. Eventually, the drug-resistant clones were selected and amplified as either G3K cells, which are viable in the presence of 3 μM gemcitabine, or T3M4-R cells, which remain viable even in the presence of 50 μM gemcitabine. These gemcitabine-resistant cells were maintained in media containing the respective final concentrations of gemcitabine (see Methods for details) prior to use in experiments. In addition, we were interested in the effects of removing the selection pressure from G3K cells over an extended period. To this end, we cultured G3K cells without gemcitabine for more than 6 months, resulting in the generation of G3K-Reverse (G3K-R) [38]. Next, the IC50 was evaluated to assess the extent of gemcitabine resistance in G3K and G3K-R cells compared to the parental Mia PaCa-2 cells, which already exhibited some degree of resistance [39]. As expected, the IC50 of gemcitabine in the parental Mia PaCa-2 cells was 1.04 ± 0.12 μM, while the G3K cells exhibited an IC50 of 73.0 ± 9.28 μM (Fig. 1A). Similarly, T3M4-R cells showed an IC50 of 521.3 ± 34.13 μM, representing a 43-fold increase of gemcitabine resistance than that of the parental T3M4 cells which had an IC50 of 12.16 ± 2.58 μM (Fig. 1B). Surprisingly, gemcitabine resistance persisted in G3K-R cells with an IC50 of 55.01 ± 4.78 μM much higher than that of Mia PaCa-2 cells. The gemcitabine cytotoxic effect was further verified by the colony formation assay (Supplemental Fig. 1A and 1B).

Since gemcitabine blocks DNA replication and induces DNA damage, we examined whether the DNA damage response was altered in G3K and G3K-R cells compared to Mia PaCa-2 cells by analyzing DNA damage checkpoint signaling. As shown in Fig. 1C, treatment with 1 μM gemcitabine for 12 h activated checkpoint signaling in G3K and G3K-R cells similarly to Mia PaCa-2 cells, as evidenced by the significant increase in phosphorylation of CHK1 at S345 and p53 at S15. These suggest that the DNA damage response signaling was not enhanced in G3K and G3K-R cells.

It has been previously reported that DNA damage promotes the formation of a mitochondria-oriented prolyl isomeric form of ATR at Ser428-Pro429 motif in the cytoplasm [35]. This form of ATR binds to tBid at the outer mitochondrial membrane, blocking the recruitment of Bax/Bak and preventing tBid-initiated apoptosis [40]. To investigate whether the interaction between the mitochondrial ATR (mtATR) and tBid contributes to DNA damaging drug resistance in cancer, cytoplasmic fractions from Mia PaCa-2, G3K and G3K-R cells were collected and analyzed by western blotting. As shown in Fig. 1D, ATR was significantly dephosphorylated at Ser428 in gemcitabine resistant cells compared to parental cells, a key event required for the generation of mitochondria-oriented isomeric ATR [35], Consistently, Duolink proximity ligation assays (PLA) showed increased mtATR-tBid complex formation at mitochondria (Fig. 1E and F). Co-immunoprecipitation assays further confirmed the interaction of mtATR with tBid (Fig. 1G). In addition, we also assessed Bid mRNA levels in G3K and Mia PaCa-2 cells. qPCR results revealed a 12-fold increase of Bid mRNA in G3K cells compared to Mia PaCa-2 cells (Fig. 1H). Similarly, T3M4-R cells also exhibited an accumulation of ATR and tBid at mitochondria compared to parental T3M4 cells (Fig. 1I).

### 2.2. The mtATR-tBid complex represents a form of apoptotic dormancy that can be reactivated by PP2A inhibition in resistant PDAC cells

tBid is a mitochondrial membrane-targeted proapoptotic protein that induces Bax/Bak oligomerization and cytochrome *c* release to trigger apoptosis [41]. Since ATR binding to tBid blocks tBid’s proapoptotic activity [35], and both antiapoptotic mtATR and proapoptotic tBid proteins accumulated at mitochondria in gemcitabine resistant PDAC cells (Fig. 1), we hypothesized the presence of tBid-mediated apoptotic dormancy in these resistant cells. We further proposed that removing mtATR from this complex could reactivate the pre-existing tBid at mitochondria and thus apoptosis.

**Fig. 2.**
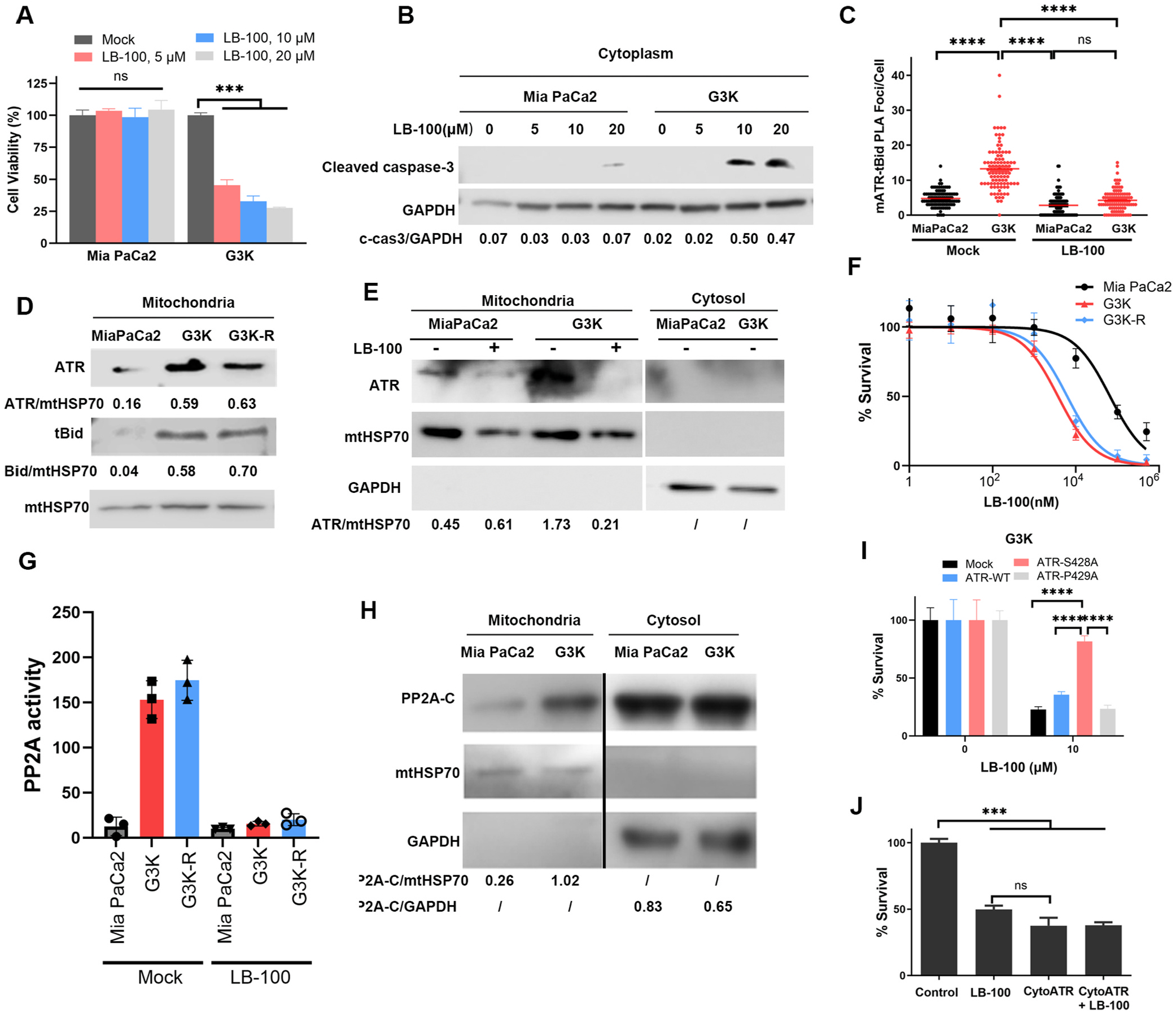
Disrupting the persistent mtATR-tBid complex at mitochondria by targeting mtATR induces apoptosis in resistant PDAC cells. (A) Cell viability was detected with methylene blue assays in Mia PaCa-2, G3K and G3K-R cells after LB-100 treatment at 3 different concentrations. Three independent experiments were performed. (B) Cytoplasm fraction of LB-100 treated Mia PaCa-2 and G3K cells were subjected to Western blot. Cleaved caspase-3 was detected. The mean values represent three independent repeats. (C) Quantification of PLA foci at mitochondria after LB-100 treatment in all three cell lines (n = 100). (D) Mitochondrial fraction was isolated from Mia PaCa-2, G3K and G3K-R cells and subjected to Western blot. ATR and Bid were detected. The mean values represent three independent repeats. (E) 10 μM LB-100 was used to treat Mia PaCa-2 and G3K cells. Mitochondrial fraction and cytosol fraction were subjected to Western blot. mtATR was detected. mHSP70 and GAPDH were used as loading control separately. The mean values represent three independent repeats. (F) Representative curves showing the cell survival of Mia PaCa-2, G3K and G3K-R cells after LB-100 treatment. The IC50 of LB-100 in the three cell lines were 66.76 ± 9.13 μM, 3.72 ± 0.59 μM and 6.21 ± 0.55 μM, from three independent measurements. (G) Mitochondrial PP2A activity was detected in Mia PaCa-2, G3K and G3K-R cells using PP2A immunoprecipitation activity assay kit. (H) PP2A-C subunit accumulation on mitochondria was compared in Mia PaCa-2 and G3K cells. Three independent measurements were performed. (I) G3K cells were transfected with full length ATR DNA harboring different mutations and challenged with 10 μM LB-100. Cell viability was detected with methylene blue assays. The experiments were repeated 3 times. (J) G3K cells were challenged with LB-100 or/and cytoATR antibody. Cell viability was determined. The experiments were repeated 3 times.

It has been shown that the formation of mtATR in the cytoplasm depends on the dephosphorylation of ATR at Ser428 by PP2A [35,37]. This dephosphorylation causes a transition of ATR from its *trans*-isomeric form, which cannot bind to tBid, to the *cis*-isomeric form (mtATR) [35,37]. Therefore, to assess whether mtATR depletion could release tBid from dormancy and reactivate apoptosis in gemcitabine-resistant cancer cells, we conducted cell survival assays by treating G3K and control Mia PaCa-2 cells, a potent PP2A inhibitor. As shown in Fig. 2A, LB-100 treatments resulted in significant cytotoxicity to G3K cells, while the parental Mia PaCa-2 cells remained insensitive to LB-100 even at 20 μM. In addition, cleaved caspase-3 was observed in LB-100 treated G3K cells at 10 μM, but little or no cleavage was detected in Mia PaCa-2 cells at the same concentration (Fig. 2B). In G3K cells, LB-100 treatment significantly reduced the accumulation of mtATR-tBid complex, while this effect was not observed in Mia PaCa-2 cells (Fig. 2C). Consistently, western blotting demonstrated that both mtATR and tBid were found to accumulate on isolated mitochondria in G3K and G3K-R cells, but not in Mia PaCa-2 cells (Fig. 2D), and LB-100 treatment abolished mitochondrial ATR (Fig. 2E). A colony formation assay also supported the sensitivity of G3K and G3K-R cells to LB-100, while parental Mia PaCa-2 cells remained unaffected (Supplemental Fig. 2A). IC50 determination further corroborated the sensitivity of gemcitabine-resistant cells to LB-100, with an IC50 of 3.72 ± 0.59 μM for G3K and 6.21 ± 0.55 μM for G3K-R cells, compared to 66.76 ± 9.13 μM for Mia PaCa-2 cells (Fig. 2F).

To verify that the elimination of mtATR by LB-100 occurred through inhibition of PP2A activity, we assessed PP2A activity using an immunoprecipitation-based PP2A phosphatase assay (MilliporeSigma). In this assay, PP2A was immunoprecipitated from mitochondria fractions isolated from Mia PaCa-2, G3K and G3K-R cells, followed by analysis of PP2A’s dephosphorylation activity. The results revealed that PP2A activity was significantly higher in G3K and G3K-R cells compared to Mia PaCa-2 cells (Fig. 2G), but significantly reduced in the presence of LB-100. This indicates that LB-100 effectively inhibited PP2A activity. Consistently, Western blot analysis of mitochondrial fractions also showed significantly higher level of PP2A in G3K cells compared to Mia PaCa-2 cells (Fig. 2H).

T3M4 and T3M4-R cells were also treated with LB-100, and the results demonstrated that T3M4-R cells became much more sensitive to LB-100, with an IC50 of 1.40 ± 0.46 μM compared to T3M4 cells with an IC50 of 6.44 ± 0.92 μM, representing a 4-fold decrease (Supplemental Fig. 2C and D), further confirming the role of PP2A in mtATR-tBid-mediated apoptotic dormancy.

To confirm that the loss of mtATR (the form of dephosphorylated ATR) was specifically linked to the LB-100-induced apoptosis in gemcitabine-resistant PDAC cells, G3K cells were transfected with a non-phosphorylatable ATR-S428A mutant (a mimic of mtATR) expression vector. The cells were then treated with LB-100, followed by cell survival assays (Fig. 2I). The results showed that while G3K cells were highly sensitive to LB-100, a mtATR-tBid-mediated collateral sensitivity, overexpression of ATR-S428A (mtATR) significantly reduced LB-100-induced cell death, supporting the role of mtATR in mediating the effects of LB-100. In contrast, transfection of the ATR-P429A mutant (which mimics *trans*-isomeric form of ATR at S428-P429 motif) had no effects. These findings further confirmed the critical role of mtATR (dephosphorylated ATR at Ser428) in preventing cells from apoptosis.

In addition, we developed and validated an antibody that specifically recognizes cytoplasmic ATR, but not nuclear ATR in cells (Supplemental Fig. 2E and F). The cytoplasmic ATR-specific antibody (cytoATR Ab) was transfected into G3K cells to neutralize cytoplasmic ATR, followed by cell survival assays. As shown in Fig. 2J, this transfection resulted in significant cell death with slightly greater effectiveness than LB-100. Combination of cytoATR Ab with LB-100 did not enhance the killing, suggesting that both the antibody and LB-100 act through the same pathway to deplete mtATR (Fig. 2J).

Taken together, our results suggest that gemcitabine-resistant PDAC cells are more sensitive to LB-100 or cytoplasmic ATR antibody, likely due to the disruption of the mtATR-tBid interaction, which reactivates tBid-mediated apoptosis.

### 2.3. Targeting mtATR synergizes with gemcitabine or FOLFIRINOX by disrupting mtATR-tBid complex in normal PDAC cells

**Fig. 3.**
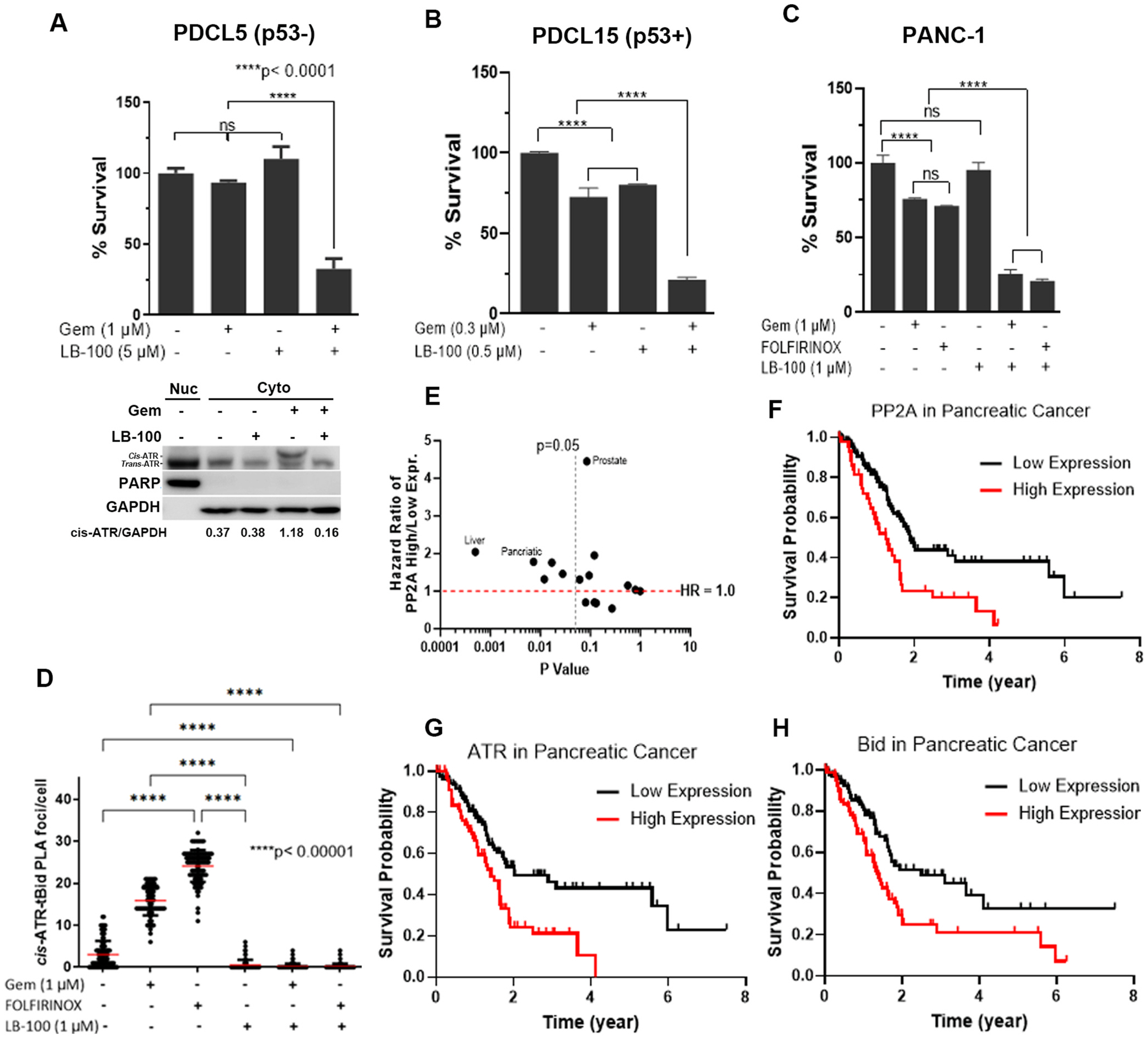
Sensitization of gemcitabine or FOLFIRINOX by mtATR antagonist PP2A inhibitor in PDAC cells and survival analysis of TCGA data. (A) and (B) Patient-derived pancreatic cancer cells, PDCL5 and PDCL15, were treated with gemcitabine and PP2A inhibitor LB-100, either alone or in combination, for 72 h, followed by MTT assays. The experiments were repeated 3 times. (C) PANC-1 cells were treated with gemcitabine or FOLFIRINOX in the presence or absence of LB-100, a selective PP2A inhibitor (a mtATR antagonist), followed by cell survival assays. The experiments were repeated 3 times. (D) PLA assays were performed to assess mtATR-tBid complex formation at the mitochondria (n = 100). (E) Multivariate hazard ratio (HR) analysis of TCGA patient data across 17 cancer types for PP2A high/low expression. (F)–(H) Kaplan-Meier survival analysis for pancreatic cancer patients, comparing survival probabilities over time for low vs. high expression of PP2A, ATR, and Bid, respectively.

In addition, we explored the potential synergy between disrupting the mtATR-tBid complex and canonical PDAC drugs in PDAC therapy. We treated two patient-derived pancreatic cancer cell lines, PDCL5 (p53-deficient) and PDCL15 (p53-WT) [42,43], with gemcitabine or FOLFIRINOX, both first-line therapies for PDAC, either alone or in combination with PP2A inhibitor LB-100. As shown in Fig. 3A and B, while gemcitabine or FOLFIRINOX alone induced minimal cell death, their combination with LB-100 significantly sensitized PDAC cells to these drugs, with a Combination Index (CI) of 0.4 [44]. This enhanced sensitivity is likely due to the inhibition of PP2A by LB-100, which prevents the dephosphorylation of ATR at S428, thus reducing the formation of mtATR. Notably, a ten-fold lower dose of LB-100 was required to achieve a similar level of synergy in p53+ PDCL15 cells compared to p53− PDCL5 cells, which aligns with the role of p53 in upregulating Bid expression in stressed cells [45].

Similar experiments were also performed with PANC-1 cells. As shown in Fig. 3C, LB-100 significantly enhanced the sensitivity of PANC-1 cells to both gemcitabine and FOLFIRINOX, although LB-100 alone had little effect. The increased sensitization was accompanied by a significant reduction in the mtATR-tBid complex to the baseline level, as measured by PLA assays (Fig. 3D). In contrast, treatment with gemcitabine or FOLFIRINOX alone resulted in a significant increase in mtATR-tBid levels (Fig. 3D), suggesting that the formation of this complex could contribute to resistance to single-drug treatments.

### 2.4. Potential clinical relevance of mtATR-tBid complex in PDAC prognosis

Given the critical role of PP2A in inducing mtATR by dephosphorylating ATR at Ser428, and to assess whether PP2A inhibition might be clinically relevant in PDAC, we analyzed TCGA patient dataset across 17 different cancer types (Human Protein Atlas). We examined the hazard ratio (HR) of high versus low PP2A expression in relation to 5-year survival probability (An HR value greater than 1.0 indicates a worse 5-year survival probability for patients with high PP2A expression compared to those with low expression). Our analysis revealed that all cancer types with a P-value <0.05 had an HR greater than 1.0, suggesting that higher PP2A expression is associated with a lower 5-year survival probability. This includes pancreatic cancer (Fig. 3E). The Kaplan-Meier plot further demonstrated a favorable outcome for patients with relatively low PP2A levels (Fig. 3F), confirming its role as a prognostic marker for pancreatic cancer (Human Protein Atlas). In line with this, an independent clinical study showed a strong correlation between the dephosphorylation of cytoplasmic p-ATR(S428) and the metastatic stages of ovarian tumors, whereas no such correlation was observed for nuclear p-ATR(S428) or p-ATM(S1981) [46].

Similar Kaplan-Meier plots were observed for ATR and Bid (Fig. 3G and H). PP2A, ATR and Bid are widely recognized as tumor suppressors. For instance, ATR kinase is well-known for its role in safeguarding genome stability against oncogenesis and carcinogenesis. However, our current analysis of clinical data suggest their involvement in cancer therapy resistance, contrasting with their canonical roles in curbing tumorigenesis. In the cases of PP2A and ATR, this resistance may be attributed to their different cellular forms and corresponding distinct functions. Specifically, the functions of PP2A are dependent on the specific isoforms of its B subunit, while ATR exists in two different isomeric forms, functioning as ATR kinase and mtATR. Although the poor survival probability of PDAC patients with high ATR expression could be associated with increased DNA repair activity during therapy, our results demonstrated that ATR kinase activity played a minimal role in the post-treatment resistance of PDAC cells (as shown later in Fig. 5). For Bid, its tumor suppressor role is well-established, as tBid is proapoptotic. However, mtATR-tBid complex-mediated apoptotic dormancy impairs tBid’s ability to carry out its function, leading to its accumulation at the mitochondria, much like the scenario seen with p53 mutants whose cellular levels are significantly elevated in cancer. For all three proteins, the clinical data suggest that their noncanonical functions play a predominant role in sustaining the survival of resistant cancer cells.

### 2.5. CIP2A degradation promotes PP2A activity on cytoplasmic ATR in G3K cells

**Fig. 4.**
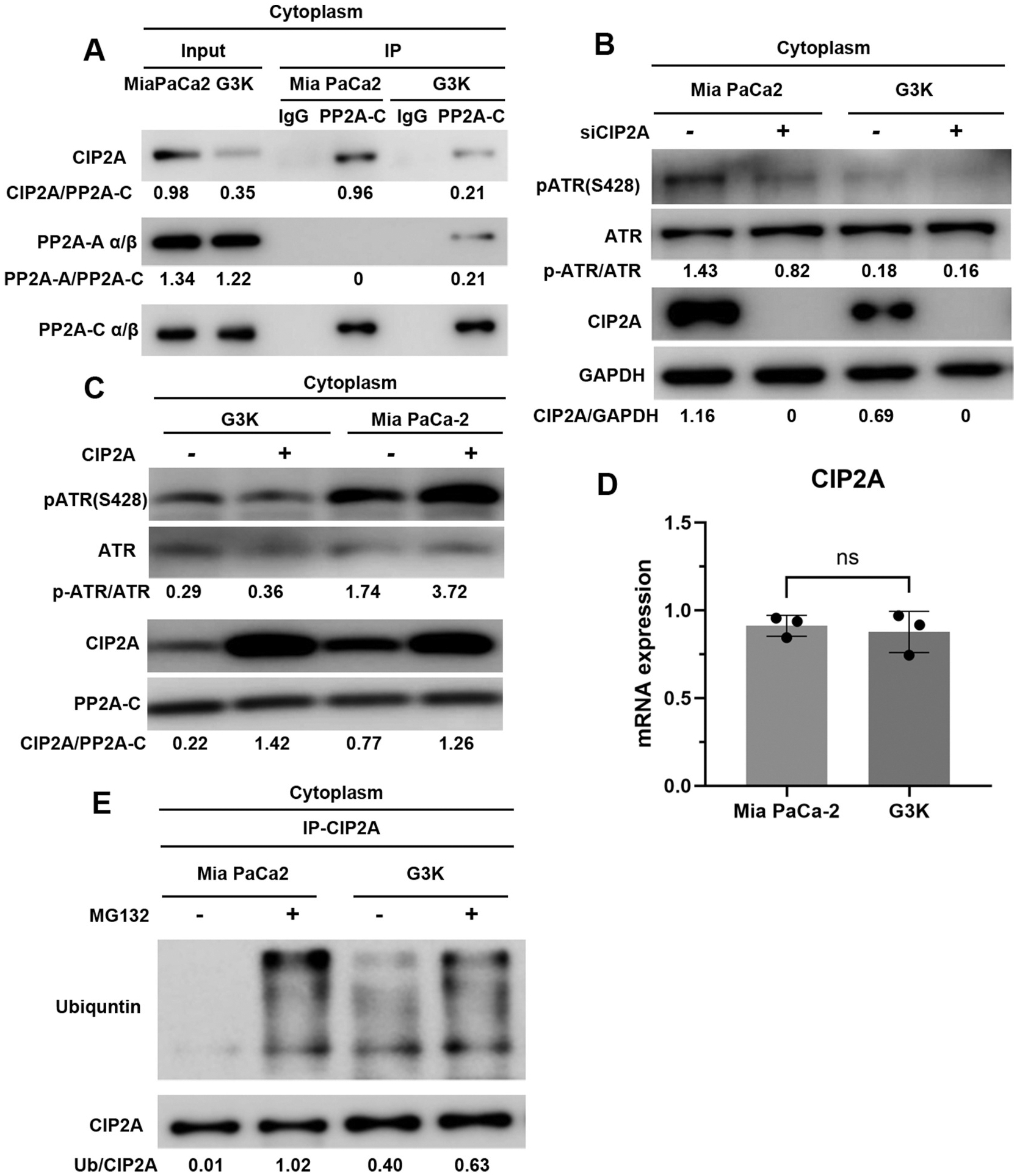
CIP2A degradation activates PP2A to dephosphorylate cytoplasmic ATR at Ser428 in gemcitabine-resistant PDAC cells. (A) Cytoplasm fraction of Mia PaCa-2 and G3K cells were subjected to PP2A-C subunit immunoprecipitation. PP2A-A subunit and CIP2A were detected. CIP2A protein was less in G3K cells. The mean values under each panel were from 3 independent repeats. (B) siRNA targeting CIP2A was transfected into Mia PaCa-2 and G3K cells. The cytoplasm fractions were subjected to Western blot. Phosphor-ATR(S428) level was lower after CIP2A knockdown. The mean values under each panel were from 3 independent repeats. (C) Overexpressing CIP2A in both Mia PaCa-2 and G3K cells upregulated Phosphor-ATR(S428) level. The mean values under each panel were from 3 independent repeats. (D) qRT-PCR was employed to detect the CIP2A mRNA level in both Mia PaCa-2 and G3K cells. The result showed no difference at the transcription level. (E) Mia PaCa-2 and G3K cells were treated with 10 μM MG132 for 2h and the cytoplasm fractions were used for CIP2A immunoprecipitation. The ubiquitin level was detected to indicate CIP2A degradation in G3K cells. The mean values under each panel were from 3 independent repeats.

To investigate the mechanisms underlying the higher PP2A activity in G3K cells compared to Mia PaCa-2, we performed co-immunoprecipitation (co-IP) by immobilizing PP2A’s catalytic subunit (PP2A-C) to identify its interacting partners in the cytoplasm, as *cis*-ATR was only present in this cellular compartment. Remarkably, as shown in Fig. 4A, cancerous inhibitor of protein phosphatase 2A (CIP2A) was significantly downregulated in G3K cells relative to Mia PaCa-2 cells (Input). Consistently, significantly less CIP2A was immunoprecipitated in G3K cells compared to Mia PaCa-2 cells, although the catalytic subunit of the PP2A holoenzyme (PP2A-C) was equally expressed (Input) and immunoprecipitated in both cell types. Notably, an opposite effect was observed for the scaffold subunit of PP2A (PP2A-A). While PP2A-A was equally expressed in the cytoplasm of both Mia PaCa-2 and G3K cells (Input) and was co-immunoprecipitated with PP2A-C in G3K cells, little or no PP2A-A was co-IPed with PP2A-C in Mia PaCa-2 cells. These results are consistent with recent reports indicating that CIP2A inhibits PP2A holoenzyme activity by displacing the PP2A-A subunit and forming a complex with PP2A-C, PP2A-B [28,47]. Specifically, the results in Fig. 4A imply that in Mia PaCa-2 cells, increased interaction between CIP2A and PP2A-C leads to greater displacement of PP2A-A compared to G3K cells, resulting in the inhibition of PP2A. In contrast, in G3K cells, the reduced CIP2A interaction with PP2A-C maintains a higher proportion of the intact PP2A holoenzyme than in Mia PaCa-2 cells, thus promoting higher PP2A activity.

To confirm that CIP2A plays a role in inhibiting PP2A-mediated dephosphorylation of ATR at Ser428, CIP2A, siRNAs were transfected into Mia PaCa-2 and G3K cells. As shown in Fig. 4B, in the absence of the siRNAs, G3K cells exhibited significantly lower phosphorylation of ATR at Ser428 compared to Mia PaCa-2 cells. This is consistent with the much lower cytoplasmic levels of CIP2A in G3K cells, suggesting higher PP2A-mediated dephosphorylation activity in G3K cells compared to Mia PaCa-2 cells. However, knockdown of CIP2A in Mia PaCa-2 cells resulted in a significant reduction of the phosphorylation of ATR at Ser428 in the cytoplasm compared to the mock transfection. A similar reduction was observed in G3K cells, although the cells already exhibited only a basal level of phosphorylation of ATR at Ser428, making the reduction less pronounced. These results are consistent with the role of CIP2A in inhibiting PP2A whose activity is required for the dephosphorylation of ATR at Ser428, and the formation of mtATR.

Given the significantly lower level of CIP2A in G3K cells compared to Mia PaCa-2 cells, G3K cells were transfected with a CIP2A expression vector. The cytoplasmic fractions of these cells were subjected to Western blot analysis. As shown in Fig. 4C, overexpression of CIP2A resulted in a significant increase in the phosphorylation of ATR at Ser428 in both Mia PaCa-2 and G3K cells, with a much greater increase observed in G3K cells. This finding supports the role of CIP2A in inhibiting PP2A, which in turn enhances ATR phosphorylation and reduces mtATR levels.

To elucidate the mechanisms underlying the downregulation of CIP2A protein levels in G3K cells, we first measured the mRNA levels of CIP2A in both Mia PaCa-2 and G3K cells using qRT-PCR, but found no significant differences (Fig. 4D). Since reactive oxygen species (ROS) have been shown to enhance CIP2A degradation and gemcitabine treatment is known to significantly increase ROS production in gemcitabine-resistant PDAC [32,48,49], we next investigated whether CIP2A was being degraded in G3K cells. To this end, both Mia PaCa-2 and G3K cells were pretreated with or without the proteasome inhibitor MG132, and the ubiquitination of CIP2A was analyzed by immunoprecipitation followed by western blotting using an anti-ubiquitin antibody. As shown in Fig. 4E, even without MG132 treatment, a high level of CIP2A ubiquitination was observed in G3K cells, while little ubiquitination was detected in Mia PaCa-2 cells. As expected, MG132 treatment significantly increased ubiquitination in both cell lines. These results suggest that the significantly reduced CIP2A protein levels in G3K cells are due to CIP2A protein degradation.

### 2.6. ATR kinase activity has no effect on the mtATR-tBid-mediated collateral sensitivity in G3K cells

**Fig. 5.**
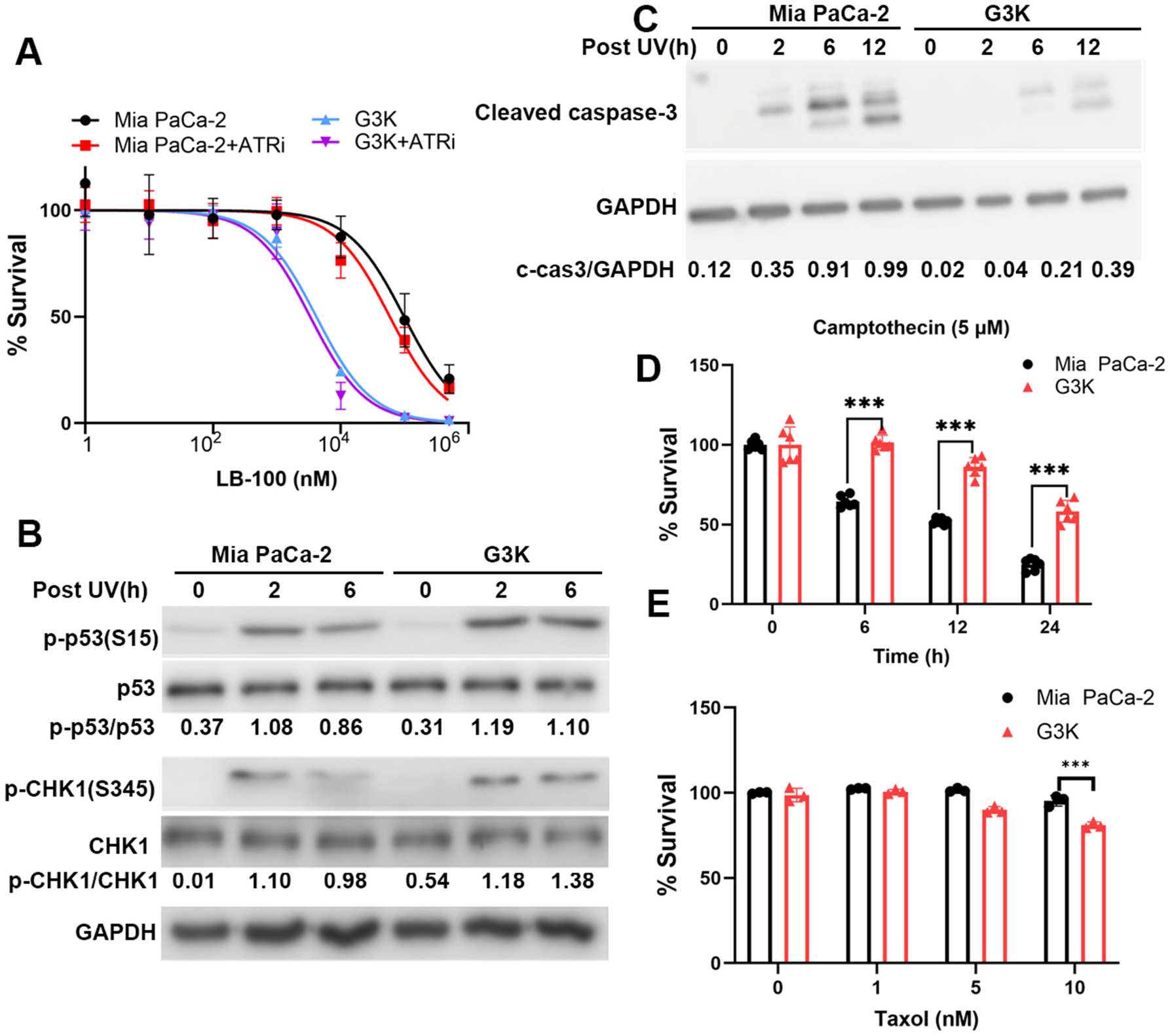
The function of mitochondrial ATR is independent of ATR kinase activity. (A) Representative cell survival curve showing the combination of LB-100 and ATR kinase inhibitor (Bay1895344, 50 nM) didn’t affect the overall killing profile of LB-100. The IC50 of LB-100 in Mia PaCa-2 and G3K cells were 114.87 ± 26.76 μM and 3.87 ± 0.31 μM. When ATR inhibitor was added, the IC50 of the two cell lines were 88.97 ± 20.11 μM and 3.75 ± 0.26 μM. The data represent 3 independent measurements. (B) Mia PaCa-2 and G3K were irradiated with 40 J/m^2^ UV and recovered for 2h and 6h. Whole-cell extracts were subjected to Western blot. Proteins involved in DDR pathway were detected. The experiments were repeated 3 times. (C) Cleaved caspase-3 was detected by western blotting in Mia PaCa-2 and G3K cells at various time points following UV irradiation. The data were from three independent experiments. (D) Mia PaCa-2 and G3K cells were treated with 5 μM Camptothecin and cell viability was determined with methylene blue assays. Three independent experiments were performed. (E) Cell viability after Paclitaxel treatment with increasing concentrations in Mia PaCa-2 and G3K cells. Three independent experiments were performed.

As a PI3K kinase protein, the role of ATR in DNA damage response signaling in the nucleus is well documented. We have previously shown that the interaction between mtATR and tBid is independent of ATR’s kinase activity [35]. To further investigate whether mtATR-tBid-mediated collateral sensitivity of G3K cells to LB-100 is dependent on ATR kinase activity, cell viability assays were performed on G3K and its parental Mia PaCa-2 cells treated with increasing concentrations of LB-100, both in the presence and absence of the specific ATR kinase inhibitor elimusertib (BAY 1895344) (Supplemental Fig. 3). As shown in Fig. 5A, there were no significant differences in G3K cell survival between mock-treated and ATRi-treated cells. The same was true for Mia PaCa-2 cells, which exhibited minimal mtATR-tBid complex formation at mitochondria. This confirms that ATR’s kinase activity is not involved in the mechanism responsible for the collateral sensitivity exhibited by the gemcitabine-resistant G3K cells to LB-100. The IC50 values of LB-100 for Mia PaCa-2 and G3K cells were changed from 114.87 ± 26.76 μM and 3.87 ± 0.31 μM to 88.97 ± 20.11 μM and 3.75 ± 0.26 μM, respectively, exhibiting no significant difference.

In addition, to examine whether the ATR kinase-independent mtATR-tBid complex formation affects ATR-dependent checkpoint signaling in general, Mia PaCa-2 and G3K cells were treated with non-gemcitabine DNA-damaging agents, UV irradiation or camptothecin (CPT). UV treatments were able to induce similar extents of DNA damage checkpoint signaling in both Mia PaCa-2 and G3K cells (Fig. 5B). However, when apoptosis was analyzed, the parental Mia PaCa-2 cells exhibited significantly higher levels of cleaved caspase 3 compared to the resistant G3K cells (Fig. 5C). Similar effects were observed in cells treated with CPT (Fig. 5D), which is consistent with the mtATR-tBid complex-mediated apoptotic dormancy in G3K cells. Remarkably, when both parental and resistant cells were treated with paclitaxel, a non-DNA-damaging anticancer drug that stabilizes microtubule and arrests cells in mitosis, there was no increased death in Mia PaCa-2 cells compared to G3K cells (Fig. 5E). In fact, the survival of Mia PaCa-2 cells was slightly higher than that of G3K cells at higher paclitaxel doses.

### 2.7. tBid is the key factor that induces apoptosis in G3K cells upon its release from mtATR-tBid complex

**Fig. 6.**
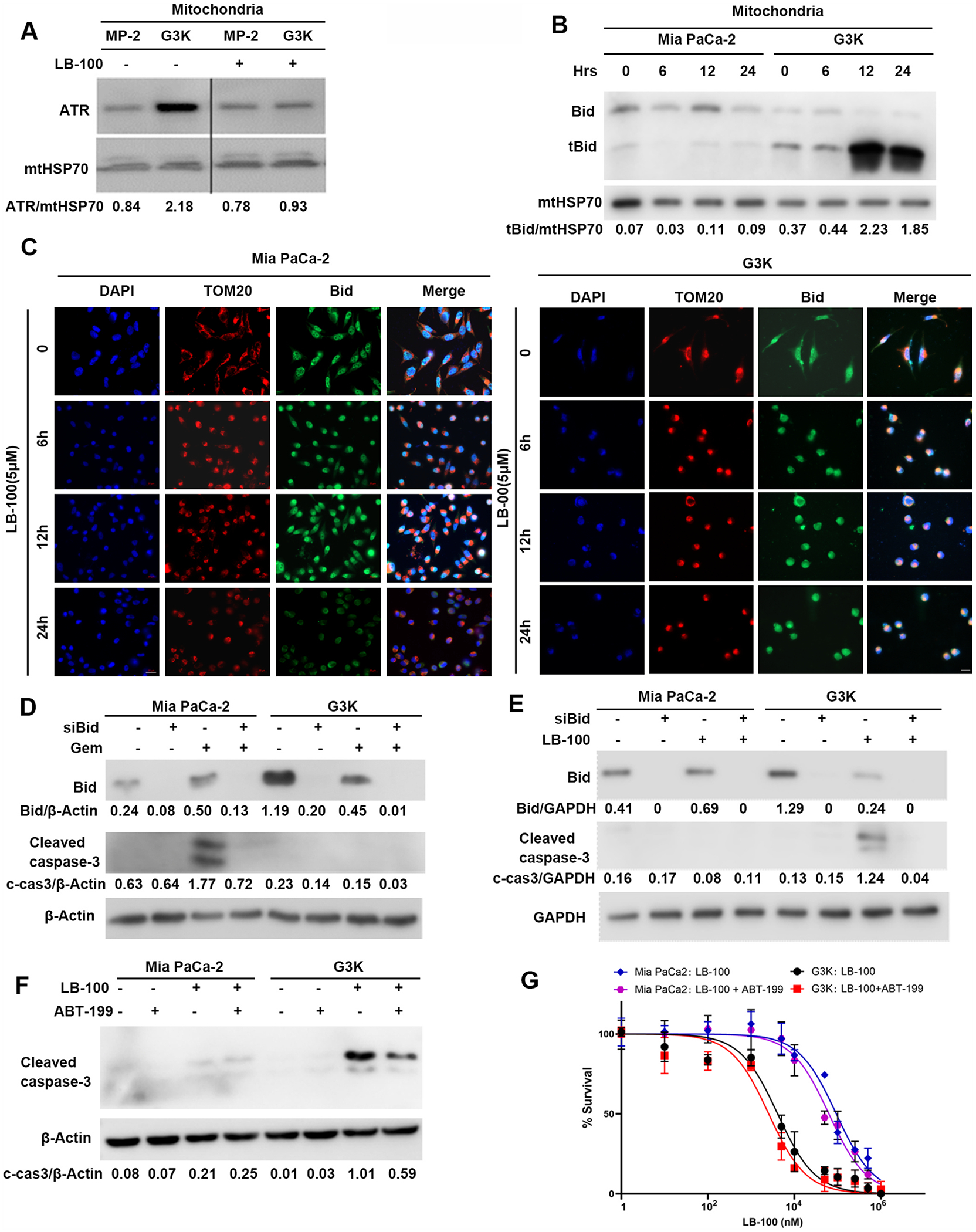
Restoring apoptosis by disrupting the mtATR-tBid complex occurs through reactivation of dormant tBid in resistant PDAC cells. (A) Mitochondrial fractions were isolated from Mia PaCa-2 and G3K cells and subjected to Western blot. LB-100 treatment resulted in ATR removal from mitochondria in G3K cells. MP-2 = Mia PaCa-2. The mean values represent three independent replicates. (B) Mia PaCa-2 and G3K were treated with 5 μM LB-100 and cells were harvested at different time points. Mitochondrial fractions were subjected to Western blot. Full length Bid and truncated Bid(tBid) were detected. The mean values represent three independent replicates. (C) Immunofluorescent results showing the Bid localization before and after LB-100 treatment. TOM20 was used as a mitochondrial indicator. Scale bar = 20 μm. The experiment was repeated 3 times. (D) Mia PaCa-2 and G3K cells were transfected with Bid siRNA and treated with 3 μM gemcitabine for 24 h. Whole cell lysate were subjected to Western blot and cleaved caspase-3 was detected. The mean values represent three independent replicates. (E) Mia PaCa-2 and G3K cells were transfected with Bid siRNA and treated with 5 μM LB-100 for 24h. Whole-cell lysates were subjected to Western blot and cleaved caspase-3 was detected. The mean values represent three independent replicates. (F) Mia PaCa-2 and G3K cells were treated with 5 nM BCL-2 inhibitor ABT-199 and 5 μM LB-100 for 24 h. Whole-cell lysates were subjected to Western blot and cleaved caspase-3 was detected. The mean values represent three independent replicates. (G) Representing IC50 curves of LB-100 in Mia PaCa-2 and G3K cells in the presence of ABT-199. The IC50 of LB-100 in Mia PaCa2 and G3K cells were 93.27 ± 6.23 μM and 4.31 ± 0.42 μM separately, while the numbers changed into 76.35 ± 7.25 μM and 2.55 ± 0.64 μM in the presence of ABT-199. Three independent experiments were performed.

Next, we investigated to confirm whether it was tBid freed from mtATR-tBid complex that triggered apoptosis in gemcitabine-resistant PDAC cells upon LB-100 treatment. tBid is the C-terminal fragment of full-length Bid, cleaved by activated caspase 8. It modulates Bak/Bax oligomerization and subsequent cytochrome *c* release [41,50]. As shown in Fig. 6A and B, mitochondrial levels of mtATR and tBid were significantly higher in G3K cells compared to Mia PaCa-2 cells. However, while LB-100 treatment of G3K cells reduced mtATR to baseline levels similar to those observed in Mia PaCa-2 cells, the mitochondrial tBid level remained unchanged after 6 h of treatment but increased dramatically after 12 h in G3K cells (Fig. 6B). The significant increase in tBid following prolonged treatment aligns with the enhanced activation of apoptosis by tBid. This observation is consistent with previous findings that tBid-mediated apoptosis activates caspases, which in turn promote further cleavage of Bid to tBid, creating a feedback loop that amplifies apoptosis in a chain reaction-like manner, but constrained by the availability of Bid [51–53].

Next, we performed immunofluorescence (IF) microscopy to analyze Bid localization in cells over time using a Bid antibody that detects both tBid and full-length Bid. As shown in Fig. 6C, in MiaPaCa-2 cells, most of the Bid was localized in the nucleus, even after 12 h of LB-100 treatment. In contrast, in G3K cells, most of Bid was already associated with mitochondria in untreated cells, and LB-100 treatment further increased this mitochondrial association over time.

To further determine the significance and specificity of tBid’s role in the mechanism of LB-100-induced apoptosis in resistant cells, Bid was knocked down with siRNA in both Mia PaCa-2 and G3K cells (Fig. 6D). As expected, knockdown of Bid abolished gemcitabine-induced apoptosis in Mia PaCa-2 cells, evidenced by the absence of cleaved caspased-3 in Mia PaCa-2 cells after the knockdown. In contrast, Bid knockdown had no effect on apoptosis in G3K cells, consistent with the presence of mtATR in these cells. However, Bid knockdown did rescue LB-100-induced apoptosis in G3K cells (Fig. 6E). Together, these results suggest that gemcitabine-induced apoptosis in parental cells and LB-100-induced apoptosis in resistant cells are both dependent on the Bid/tBid activity.

To determine whether abolishing the antiapoptotic mtATR is exclusively responsible for the LB-100-induced apoptosis in G3K cells or whether other canonical antiapoptotic pathways were also involved, ABT-199, a highly selective inhibitor of antiapoptotic Bcl-2 protein, was used in combination with LB-100 treatment. As shown in Fig. 6F, inhibiting Bcl-2 had no or little effect on the LB-100’s action. The IC50 values for the sensitivity of both Mia PaCa-2 and G3K cells to LB-100 were 93.27 ± 6.23 μM and 4.32 ± 0.42 μM, respectively, in the presence of inhibitor ABT-199. In contrast, the IC50 values without the inhibitor were 76.35 ± 7.25 μM and 2.55 ± 0.64 μM, with no significant differences analyzed by *t*-test (Fig. 6G).

### 2.8. Antagonizing mtATR by LB-100 significantly reduced gemcitabine-resistant PDAC tumor growth in vivo

To determine whether the mtATR-tBid mediated collateral sensitivity to LB-100 in G3K cells could affect tumor growth *in vivo*, we orthotopically implanted G-luciferase-tagged G3K xenograft tumors into the pancreas of athymic nude mice. The mice were randomly assigned to control and LB-100-treated groups, and tumor growth was monitored over time measuring bioluminescence through blood sampling twice weekly.

**Fig. 7.**
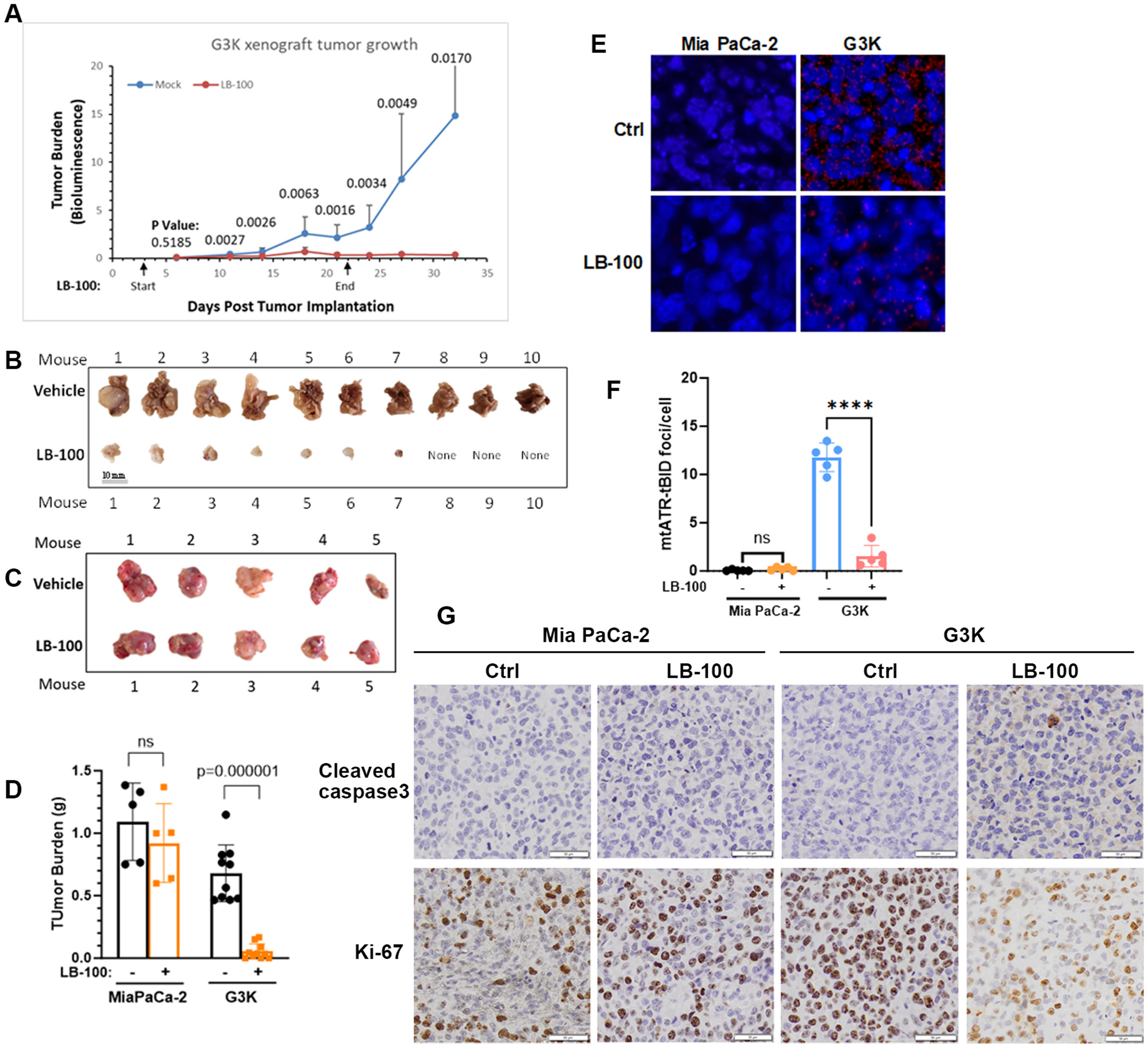
PP2Ai-medicated targeting of mtATR significantly reduces tumor burden in gemcitabine-resistant orthotopic PDAC xenografts. (A) Bioluminescent assay detecting the tumor burden after G3K cell xenograft with or without LB-100 administration. (B) Representative G3K xenograft tumors. (C) Representative Mia PaCa-2 xenograft tumors. (D) Quantification of tumor weight. Mia Paca-2 tumor xenografts: 5 male nude mice; G3K tumor xenografts: 10 male nude mice. (E) PLA assay to detect the ATR-tBid foci in xenograft tumor samples. (F) Quantification of PLA foci. Average foci of each cell were quantified from five different tumors. (G) Representative IHC staining of cleaved caspase-3 and Ki67 from five Mia PaCa-2 and five G3K xenograft tumor samples with or without LB-100 administration. Scale bar = 50 μm.

As shown in Fig. 7A, after G3K cell transplantation, tumors in the mock treatment group continued to grow steadily, as evidenced by the increasing bioluminescence signal. However, in mice treated with LB-100, tumor growth was dramatically reduced, with some tumors becoming undetectable. These findings were further confirmed by the tumor burdens harvested at the end of the study (Fig. 7B). In contrast, LB-100 treatment didn’t significantly reduce tumor burden in Mia PaCa-2 cell-transplanted mice (Fig. 7C). Quantification of the *in vivo* results is shown in Fig. 7D. These data suggested that similar to the *in vitro* findings, G3K pancreatic tumor xenografts exhibit collateral sensitivity to LB-100 *in vivo* leading to significant inhibition of tumor growth.

In addition, we performed PLA analysis to assess ATR-tBid interaction in the tumors. Consistent with the *in vitro* results, LB-100 treatment significantly reduced ATR-tBid interaction in G3K tumors, further supporting the role of this interaction in the growth of gemcitabine-resistant G3K cells (Fig. 7E and F). In contrast, little or no foci were observed in Mia PaCa-2 xenografts, regardless of LB-100 treatments (Fig. 7E and F). Finally, immunohistochemistry (IHC) staining revealed differences in Ki67 expression in the xenografts. Both Mia PaCa-2 and G3K tumors from mock-treated mice showed high levels of Ki67, indicating active malignant tumor growth. However, in G3K tumors, Ki67 staining was significantly reduced following LB-100 treatment, while Mia PaCa-2 tumors showed minimal change. Correspondingly, cleaved-caspase 3 levels were elevated in G3K tumors after LB-100 administration, suggesting an increase in cell death (Fig. 7G).

## 3. Discussion

Using acquired drug-resistant cancer cell lines to study resistance mechanisms offers several advantages by minimizing or eliminating confounding factors that can complicate result interpretation in other models, such as selective cytotoxic effects across different cell lines [54–56] and variability in efficacy among individual patients [57]. This controlled approach allows a focused analysis of the specific mechanisms driving resistance, without the interference of pre-existing cellular heterogeneity, tumor microenvironment variability, immune system effects, or off-target drug interaction. In this study, two groups of acquired PDAC resistant cell lines, Mia PaCa-2 versus G3K, and T3M4 versus T3M4-R, were established to study the mechanisms of PDAC gemcitabine resistance.

Here, we present evidence for a novel apoptotic dormancy-based resistance mechanism, which is induced by CIP2A degradation-mediated PP2A activation within the CIP2A-PP2A-mtATR-tBid signaling axis. Our understanding of this mechanism provides an alternative strategy for overcoming resistance in cancer cells, bypassing conventional challenges such as DNA repair pathways and drug efflux. This strategy focuses on releasing the pre-accumulated proapoptotic tBid from its dormant state within the mtATR-tBid complex by targeting PP2A or cytoplasmic ATR, the precursor of mtATR, leveraging insights from this newly defined drug resistance mechanism.

As a truncated C-terminal fragment of Bid, the proapoptotic protein tBid plays a crucial role in activation of mitochondria-mediated apoptosis [58]. Similar to canonical antiapoptotic Bcl-2 proteins, such as Bcl-2, Bcl-XL, Bcl-W, and MCL-1, mtATR is also an antiapoptotic protein translocated to mitochondria. However, unlike these antiapoptotic Bcl-2 proteins, which bind and inhibit Bax/Bak to prevent apoptosis, mtATR does not interact directly with Bax/Bak [35]. Instead, mtATR binds tBid to form a complex that directly inhibits tBid-mediated activation of Bax/Bak, thereby preventing the release of cytochrome *c* [35]. In established conventional mechanisms, tBid binds to Bcl-2 proteins to disrupt their interaction with Bax/Bak, thus freeing Bax/Bak, while simultaneously interacting with Bax/Bak to promote their oligomerization. Therefore, mtATR may represent a novel type of antiapoptotic protein that plays a direct role in regulating apoptosis. The results from this study highlight the importance of mtATR-tBid complex in DNA damage-based cancer drug resistance.

Our results highlight the potentially distinct roles of PP2A and ATR as tumor promoters versus suppressors in the contexts of carcinogenesis and cancer therapy. While PP2A is generally considered a tumor suppressor, it plays a critical role in many cellular processes essentially for either cancer development or treatment [25,30,34,59–62]. Since the formation of mtATR depends on the dephosphorylation of cytoplasmic ATR at Ser428 by PP2A, the antiapoptotic role of mtATR in cancer drug resistance positions PP2A as a promising target for overcoming resistance in cancer therapy. As demonstrated in this study, the PP2A-specific inhibitor LB-100 eliminates mtATR from mitochondria, leading to the release of tBid and the reactivation of apoptosis. Notably, apoptosis occurs only in resistant PDAC cells, not in parental PDAC cells. Consistently, PP2A activity towards cytoplasmic ATR is significantly higher in resistant PDAC cells than in parental, non-resistant cells. Furthermore, apoptosis in the resistant cells is specifically triggered by the reactivation of tBid induced by LB-100, whereas inhibition of the antiapoptotic Bcl-2 proteins has only limited effects (Fig. 6).

Interestingly, our further investigation reveals that the elevated PP2A activity in resistant cells is linked to the degradation of CIP2A, an inhibitory binding partner of PP2A. CIP2A is generally considered an oncoprotein due to its role in inhibiting PP2A, which aligns with PP2A’s conventional tumor-suppressive function. However, this study shows that CIP2A undergoes extensive ubiquitination-mediated degradation in resistant PDAC cells, mirroring the role of PP2A in gemcitabine resistance as evidenced in this study. This is surprising, as targeting CIP2A has been shown to enhance PDAC sensitivity to gemcitabine and other DNA-damaging agents [63–65]. A possible explanation for this discrepancy is that while CIP2A targeting sensitizes parental PDAC cells with high CIP2A levels to gemcitabine, as seen in Mia PaCa-2 cells, resistance eventually develops due to CIP2A degradation. Although the exact cause of CIP2A degradation in resistant PDAC cells remains unclear, previous studies suggest that elevated mitochondrial reactive oxygen species (mROS) may drive CIP2A degradation [32], and gemcitabine is known to increase mitochondrial ROS [48].

LB-100, either alone or in combination with other drugs, has been evaluated in clinical trials for treating small cell lung cancer, ovarian carcinoma, glioblastoma, and myelodysplastic syndromes. The safety and tolerability of LB-100 in these trials have supported its continued investigation [66]. However, these trials are based on the premise that PP2A inhibition disrupts cell division and drives senescent cancer cells into mitosis, promoting cell death via mitotic catastrophe [66,67]. In contrast, our study uncovers a novel mechanism by which LB-100 induces cell death in resistant PDAC cells through the PP2A-mtATR-tBid axis. *In vivo studies* further confirm that LB-100 induces mitochondria-mediated apoptosis exclusively in resistant PDAC cells (Fig. 7). In addition, even for PDAC cells that are not fully resistant, low doses of LB-100 significantly sensitize them to gemcitabine or FOLFIRINOX. Nevertheless, our results also suggest that while LB-100 is a promising PP2A inhibitor for cancer therapy, directly targeting mtATR, as illustrated by the use of an anti-cytoATR antibody, may offer a more beneficial strategy by minimizing adverse and non-specific effects.

Finally, given that the induction of mtATR in response to DNA damage has been observed in different types of cancer and non-cancer cells [35], it is plausible that the mtATR-tBid complex may also contribute significantly to drug resistance of other cancer types in DNA damage-based therapies. Our findings suggest mtATR as a potentially important target for overcoming resistance in cancer therapy.

## 4. Materials and Methods

### 4.1. Cell and cell culture

Mia PaCa-2, G3K and G3K-R cells as well as T3M4 and T3M4-R cells were kindly provided by Dr. Jiang-Ting Zhang in the University of Toledo. To generate gemcitabine resistant PDAC, Mia PaCa-2 cells were cultured in gemcitabine supplemented culture medium with increasing concentration with 3 μM as the final concentration. To generate G3K reverse(G3K-R) cells, the G3K cells were cultured in complete medium without gemcitabine for over 6 months. High glucose DMEM supplemented with 10 % FBS as well as 2.5 % horse serum (all from Hyclone) was used to culture Mia PaCa-2, G3K and G3K-R cells. T3M4 and T3M4-R cells were cultured with high glucose DMEM with only 10 % FBS. G3K cells were cultured in the presence of 3 μM gemcitabine while 50 μM for T3M4-R cells. To treat cells with LB-100, all cell lines were cultured with serum-free DMEM medium for 24 h and LB-100 was added for another 24h. For UV irradiation, 40 J/m^2^ UV was irradiated with a UV lamp. After UV treatment, cells were allowed for recovery for 2h.

### 4.2. Sub-cellular fractionation and western blotting

All drugs mentioned in our study are from Sigma otherwise mentioned. To fractionate cytoplasm and nucleus, cells were trypsinized and washed with PBS twice. Cytolysis buffer (3 mM CaCl_2_, 10 mM KCl, 10 mM HEPES pH 7.9, 0.34 M Sucrose, 10 % glycerol, 0.1% Triton X-100) supplemented with 1 mM DTT lysed cells in ice for 10min, followed by centrifuge at 2300 rpm for 7 min under 4 °C. The supernatant was the cytoplasm fraction. The pellet was washed with cytolysis buffer with 30 mM DTT twice, centrifuging at 2300 rpm for 7 min. Next the pellet was lysed with AB buffer (50 mM Tris-HCl, PH7.8, 140 mM NaCl, 0.5% NP-40, 1 mM EDTA, 10 % glycerol, 1% Triton X-100) for 20min in ice, followed by centrifuge at 10,000 rpm for 10 min at 4 °C. Protease inhibitor (ThermoFisher, 78428) was added in both buffers.

To isolate mitochondria, mitochondria isolation kit (Qiagen, 37612) was used. The protocol was following the kit manual, with the exception that 3 mM CaCl_2_ was added in both lysis buffer and interruption buffer. 20–30 μg protein lysate was used to perform Western blot. SDS-PAGE gel running, and membrane transfer steps were universal protocol. The only exception was that the 30 V, 180 min transfer method was used to detect ATR. For all the specific antibodies used for western blotting (Table 1).

### 4.3. PLA assay, immunofluorescence and foci quantification

In order to detect the direct interaction between ATR and tBid, a proximity ligand assay was employed. Duolink^®^ In Situ Detection Reagents Red (Sigma, DUO 92008), Duolink^®^ In Situ PLA^®^ Probe Anti-Rabbit PLUS (sigma, DUO92002), and Duolink^®^ In Situ PLA^®^ Probe Anti-Mouse MINUS (Sigma, Duo 92004) were used in our experiments. Cells were fixed with 4 % PFA for 15 min, and permeabilized with 0.5 % Triton in PBS for 10min. The remaining steps were following the protocol. The final step was to label the mitochondria with mtHSP70 antibody. To perform immunofluorescence microscopy, cells growing on cover slides were fixed with 4 % PFA, followed by 0.5 % Triton X-100 in PBS for 10 min to permeabilize cells. The cells were fixed with the blocking buffer provided in the PLA assay kit. Primary and secondary antibodies usage information are listed in Table 1.

To quantify the PLA foci, Image J (Fiji version) was used to quantify the red foci as well as the nucleus. The average number of PLA foci per cell was used to get the final quantification data.

### 4.4. Cell survival assay

Methylene blue was used to detect the cell survival rate. In general, the attached cells in 96 well plate were washed with PBS twice. 100 % cold methanol was applied to each well to fix cells for more than 30 min. Then 1 % methylene blue in 10 mM Borate buffer (pH 8.5) was used to stain fixed cells for up to 2 h. Cells were then washed with 10 mM Borate buffer thoroughly (>3 times). The plate was air dried in the fume hood. Cells were then lysed with 100 μL lysis buffer (50 % 0.1N HCl and 50 % ethanol). After there were no visible cells in the plate, the color absorbance was examined on a plate reader (SpectraMax iD5) at OD 650 with Softmax pro 7 software. GraphPad 9.0 was used to do the quantification and calculate the IC50 and cell survival rate.

### 4.5. Animals

All animal protocols were approved by IACUC of the University of Toledo. To do xenograft, Mia PaCa-2 and G3K cells were mixed with 50 % Matrigel (corning) and a total of 1 million cells were injected into the pancreases of male athymic nude mice (NU/J, JAX, 002019). For LB-100 injection, randomly grouped mice were injected with 2 mg/kg started on day 3 after surgery. Then the injection was administrated every other day for 6 times. Beginning at the 7th injection, the LB-100 dosage was reduced to 1.5 mg/kg, which lasted 5 times. On day 28 after surgery, the mice were sacrificed with carbon dioxide and the tumors at the pancreatic area were removed and fixed with formalin for further paraffin embedding. To monitor the tumor growth, 5 μL blood was collected from the mouse tail and transferred into 100 μL PBS. After centrifuging at 400 rpm for 10 min, 50 μL supernatant was transferred to a black 96-well for the Luminoskan Ascent plate reader, which pumped the same amount of Gluc reagent working solution (Nanolight technology) into each well and read the luminescence value. Higher value meant larger tumor. The final value was plotted with GraphPad 9.0.

### 4.6. PP2A phosphatase activity measurement

PP2A immunoprecipitation phosphatase activity kit (Sigma, 17–313) was used to detect the PP2A activity in cellular fractions. The protocol strictly followed the product manual. Briefly, the mitochondria were resuspended in the phosphatase buffer and mixed with 4 μL PP2A-C antibody as well as protein A agarose beads. After rotating for 4h to overnight, the beads were washed with TBST 3 times and phosphatase buffer once. 60 μL diluted phosphopeptide and 20 μL phosphatase buffer were added into the tube. The beads were incubated for 10 min under 30 °C. Next, 25 μL supernatant was transferred into the microtiter plate to mix with 100 μL Malachite Green phosphate detection solution. Let color develop for 15 min at room temperature. The plate was read at OD650.

### 4.7. DNA, siRNA and antibody transfections

The Lipofectamine 3000 transfection reagent (ThermoFisher, L3000008) and LipofectamineTM RNAiMax (ThermoFisher, 13778075) were used to transfect DNA and siRNA, respectively. The transfection was strictly following the product manual. 5 μg DNA or 5 nM siRNA were used for transfection in a 10 cm dish with 70% cellular confluency.

To transfect cyto-ATR antibody into G3K cells, Xfect^™^ Protein Transfection Reagent (TaKaRa,631325) was used. Transfection strictly followed the reagent manual, and 0.25 μg antibody was used for a 3.5 cm dish. To show the transfection was successful, the transfected cells were fixed and permeabilized and probe with anti-rabbit Alexa Fluro 488 (ThermoFisher) and subjected under fluorescent microscope. Fluorescent signal identified in cytoplasm indicated antibody transfection. Mock transfected cells were used as undetectable control.

### 4.8. Tumor paraffin section and immunohistology

Paraffin embedded tissue slides were deparaffinized with xylene (Sigma, 534056) for 10min, followed by rehydrating the tissues with 2 min 100% ethanol, 2 min 95% ethanol, 2 min 70% ethanol and 5 min H_2_O. Then the antigen was retrieved by boiling slides in 10 mM citrate acid buffer, pH 6.0 for 20 min and then cooled down naturally. The slides were incubated with 3% H_2_O_2_ solution for 15 min in dark to quench the endogenous peroxidase activity. Wash the slides with washing buffer (TBST with 0.25% Triton X-100) 3 times, 5 min each. The tissues were incubated with blocking buffer (10% FBS in washing buffer) for 2 h. Corresponding primary antibody was diluted in blocking buffer and incubate the slides at 4 °C overnight. Secondary antibodies were conjugated with biotin. After 1 h secondary antibodies probing, streptavidin conjugated HRP (ThermoFisher, SA10001,1:200) was used to further culture slides for 30 min at room temperature. 3,3′-Diaminobenzidine tablets (Sigma, D4293) were used to develop signals. Then the slides were immersed into hematoxylin (Sigma, HHS16) for nuclear staining. After differentiation and counterstaining, the tissues were dehydrated with 2 min 70% ethanol, 2 min 95% ethanol, 2 min 100% ethanol and 5 min xylene. The slides were mounted with DPX mountant for histology (Sigma, 06522).

### 4.9. Statistical analysis

All experiments were repeated at least three times. Statistical analyses were performed using GraphPad Prism 10. Quantitative data are presented as MEAN ± SD for IC_50_ values and associated figures, and as MEAN ± SEM in bar graphs, based on a minimum of three biological replicates. Statistical significance between two groups was assessed using a Student’s *t*-test, while one-way ANOVA was applied for comparisons involving more than two groups. A *p*-value <0.05 was considered statistically significant.

To determine the synergy (Combination Index CI value) of gemcitabine and LB-100 combination treatments in cancer cells, the following formula was used:

$$CI=\left(\frac{C_{gem}}{IC50_{gem}}\right)+\left(\frac{C_{LB}}{IC50_{LB}}\right)$$

Where C_gem_ and C_LB_ are the concentrations of gemcitabine and LB-100, respectively, which result in 50% survive of cancer cells when the drugs are combined. IC50_gem_ and IC50_LB_ stand for the IC50 values of gemcitabine and LB-100, respectively, when the drugs are administered individually [44].

## Supplementary Material

1

## Figures and Tables

**Table 1:** Antibodies.

Antibody	Resource	Usage
ATR	Bethyl Laboratories A300–137A, A300–138A	WB 1:10000; PLA 1:250
BID	Santa Cruz Biotechnology SC-373939	WB 1:500; PLA 1:75
CHK1	Cell Signaling 2360	WB 1:1000
p-CHK1(S345)	Cell Signaling 2348	WB 1:1000
CHK2	Cell Signaling 2334	WB: 1:2000
p53	Cell Signaling 2524	WB 1:1000
p-p53(S15)	Cell Signaling 9284	WB 1:1000
GAPDH	Santa Cruz Biotechnology SC-32233	WB: 1:10000
β-ACTIN	Santa Cruz Biotechnology SC-47778	WB 1:5000
mHSP70	Millipore MABS1955	IF 1:200; WB 1:1000
p-ATR(S428)	Cell Signaling 2853	WB 1:1000
PP2A C subunit	Cell Signaling 2259	WB 1:1000
Ki-67	Cell Signaling 9449	IHC 1:1000
Cleaved caspase 3	Cell Signaling 9661	WB 1:1000
PARP	Cell Signaling 9532	WB 1:1000
Alexa Fluor^™^ 488	ThermoFisher A11029	IF: 1:1000
Goat Anti-Rabbit IgG (H + L) 594	Biotium 20112	IF 1:1000
PP2A A subunit	Santa Cruz biotechnology SC-74580	WB: 1:1000
CIP2A	Santa Cruz biotechnology SC-80659	WB: 1:1000
Ubiquitin	Cell Signaling 20326	WB: 1:1000
pATR-T1989	Cell Signaling 30632	WB: 1:1000

## Appendix A. Supplementary data

Supplementary data to this article can be found online at https://doi.org/10.1016/j.canlet.2025.217790.
