# Supplementary material for: A PP2A-mtATR-tBid axis links DNA damage-induced CIP2A degradation to apoptotic dormancy and therapeutic resistance in PDAC
Source: Cancer Lett. Author manuscript; Available in PMC 2026 Jun 9. (PMC13249146; doi:10.1016/j.canlet.2025.217790)
Supplement: 1 [file NIHMS2175675-supplement-1.pdf]

Supplementary Figure 1

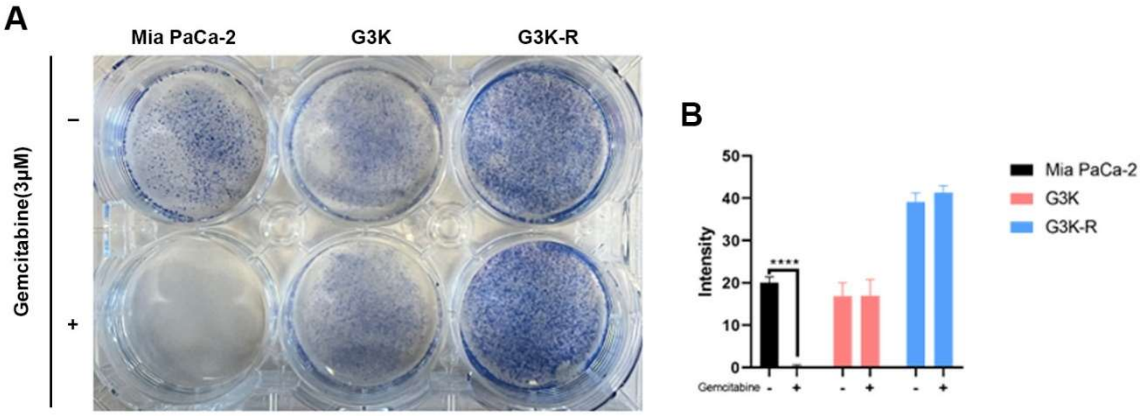

Supplementary Figure 1. Cell growth assay of Mia PaCa-2, G3K and G3K-R cells treated with 3 μM gemcitabine for 72h. Then gemcitabine was removed to let the cells grow for another 7 days. (B) Quantification of cell density by Image J.

Supplementary Figure 2

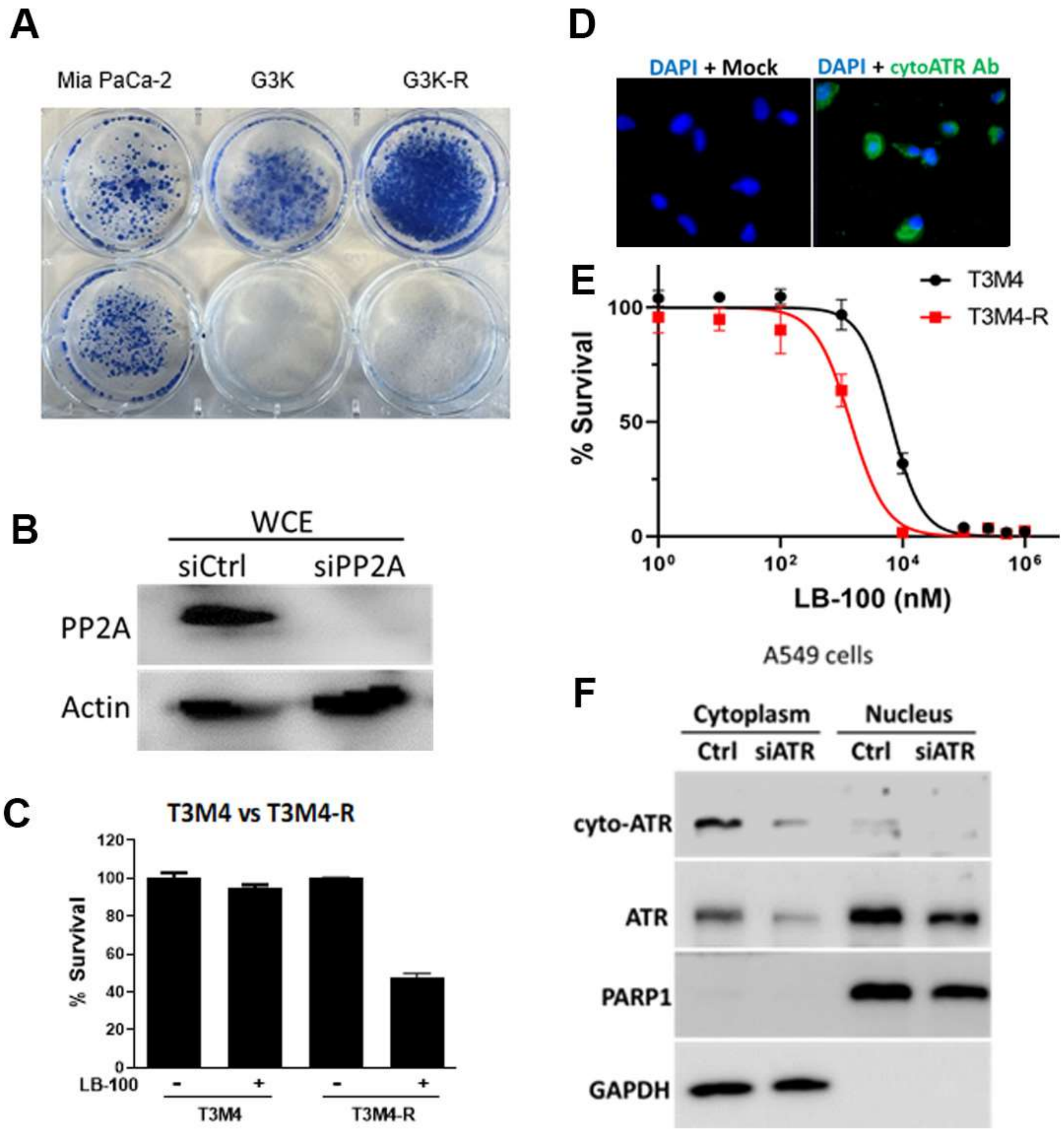

Supplementary Figure 2. (A) Cell growth assay after 5 µM LB-100 treatment for 24 h, followed by another 7 days growth in complete medium. (B) Western blot to detect the PP2A siRNA efficiency in G3K cells. (C) Cell survival rate of T3M4 and T3M4-R cells treated with LB-100. (D) Repetitive cell survival curves with LB-100 treatment in T3M4 and T3M4-R cells. The IC50 of the two cells lines were 13.4µM and 5.6µM. (E) Representative figures showing cyto-ATR antibody transfection efficiency. Green signals around the nucleus (DAPI staining) were the transfected antibody. (F) A549 cells were transfected with ATR siRNA. Nucleus and cytoplasm fractions were used for western blot. Cyto-ATR antibody only detected ATR in cytoplasm fractions.

Supplementary Figure 3

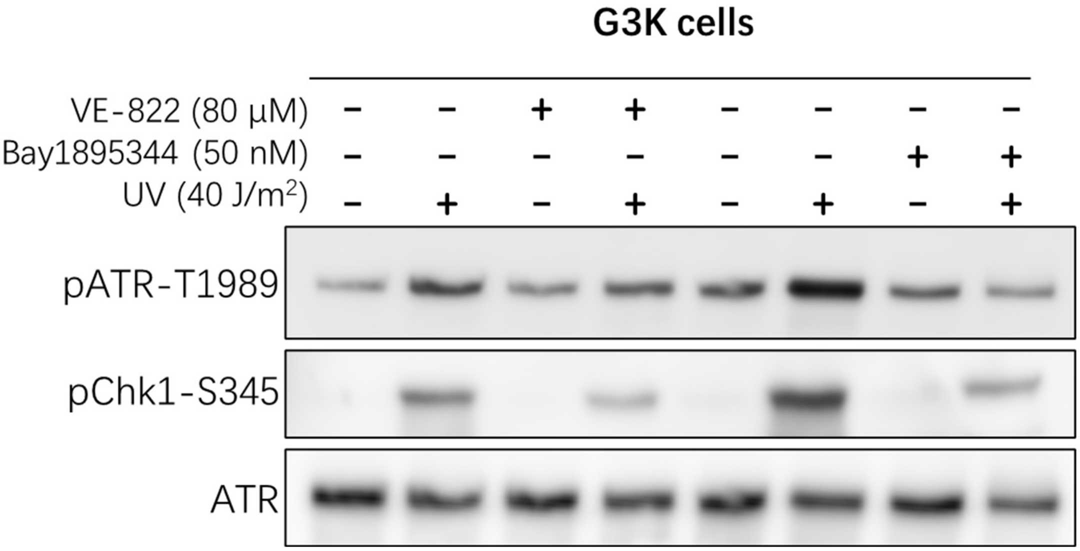

Supplementary Figure 3. G3K cells were irradiated with 40 J/m^2 UV and treated with two kinds of ATR kinase inhibitors for 2h. Whole cell lysates were subjected to western blot. The result showed that Bay 1895344 was efficiently inhibiting ATR activity as evidenced by the decreased expression of phosphorylated ATR at T1989 and phospho-CHK1 at S345.
